# Supplementary material for: From miRNA Target Gene Network to miRNA Function: miR-375 Might Regulate Apoptosis and Actin Dynamics in the Heart Muscle via Rho-GTPases-Dependent Pathways
Source: Int J Mol Sci. 2020 Dec 18;21(24):9670. doi: 10.3390/ijms21249670 (PMC7765785; doi:10.3390/ijms21249670)
Supplement: Supplementary file 1 [file ijms-21-09670-s001.zip › ijms-995615-supplementary.docx]

Table S1. The nodes and its centrality in the largest connected component of the miR-375 target gene interaction network. All genes presented are expressed in the heart muscle.

| **Node label** | **Gene Symbol** | **Centrality** |
| --- | --- | --- |
| PIK3CA | *PIK3CA* | 0,498 |
| RHOA | *RHOA* | 0,488 |
| MAPK3 | *MAPK3* | 0,485 |
| PAFAH1B1 | *PAFAH1B1* | 0,383 |
| CTNNB1 | *CTNNB1* | 0,362 |
| MYC | *MYC* | 0,361 |
| PRKCA | *PRKCA* | 0,301 |
| ERBB2 | *ERBB2* | 0,287 |
| CDC42 | *CDC42* | 0,283 |
| 1 | *YWHAZ* | 0,268 |
| 2 | *MAPKAP1* | 0,149 |
| 3 | *DEPTOR* | 0,133 |
| 4 | *CHPT1* | 0,115 |
| 5 | *TFRC* | 0,102 |
| 6 | *JAK2* | 0,086 |
| 7 | *RAB10* | 0,077 |
| 8 | *RPGRIP1L* | 0,077 |
| 9 | *UGCG* | 0,077 |
| 10 | *BCL2L11* | 0,077 |
| 11 | *CUL5* | 0,077 |
| 12 | *IFIT1* | 0,077 |
| 13 | *NUMB* | 0,077 |
| 14 | *FZD4* | 0,077 |
| 15 | *IGF1R* | 0,062 |
| 16 | *ARHGDIA* | 0,061 |
| 17 | *EFNB2* | 0,058 |
| 18 | *PAFAH2* | 0,058 |
| 19 | *LPCAT1* | 0,058 |
| 20 | *LRP5* | 0,044 |
| 21 | *RRAGB* | 0,038 |
| 22 | *SP1* | 0,038 |
| 23 | *ARHGAP11A* | 0,038 |
| 24 | *RRAGC* | 0,038 |
| 25 | *TNS1* | 0,038 |
| 26 | *ENAH* | 0,038 |
| 27 | *EXOC6* | 0,019 |
| 28 | *EIF2S1* | 0,019 |
| 29 | *BAK1* | 0,019 |
| 30 | *UBE3A* | 0,019 |
| 31 | *PRDX3* | 0,019 |
| 32 | *DUSP6* | 0,019 |
| 33 | *SKA2* | 0,019 |
| 34 | *DGKD* | 0,019 |
| 35 | *MST1R* | 0,019 |
| 36 | *PEBP1* | 0,019 |
| 37 | *PSME4* | 0,019 |
| 38 | *SGMS2* | 0,019 |
| 39 | *AP3B1* | 0,019 |
| 40 | *CEP70* | 0,019 |
| 41 | *MTDH* | 0,019 |
| 42 | *PTPN12* | 0,019 |
| 43 | *IFI6* | 0,019 |
| 44 | *AXL* | 0,019 |

Table S2. Reactome overrepresentation analysis for the key miR-375 target genes.

| **Pathway name** | **p-value** | **Overrepresented genes** |
| --- | --- | --- |
| RUNX3 regulates WNT signaling | 0,000015 | *MYC,CTNNB1* |
| Binding of TCF/LEF:CTNNB1 to target gene promoters | 0,000015 | *MYC,CTNNB1* |
| VEGFA-VEGFR2 Pathway | 0,000365 | *CDC42,PIK3CA,CTNNB1,PRKCA,RHOA* |
| Signaling by ERBB2 | 0,000463 | *PIK3CA,ERBB2,PRKCA,RHOA* |
| Signaling by VEGF | 0,000523 | *CDC42,PIK3CA,CTNNB1,PRKCA,RHOA* |
| Ca2+ pathway | 0,000533 | *MYC,CTNNB1,PRKCA* |
| Beta-catenin independent WNT signaling | 0,000986 | *MYC,CTNNB1,PRKCA,RHOA* |
| GPVI-mediated activation cascade | 0,001039 | *CDC42,PIK3CA,RHOA* |
| RHO GTPases activate KTN1 | 0,001419 | *CDC42,RHOA* |
| PI3K/AKT activation | 0,001660 | *PIK3CA,RHOA* |
| Transcriptional regulation by the AP-2 (TFAP2) family of transcription factors | 0,001784 | *MYC,ERBB2* |
| Repression of WNT target genes | 0,002493 | *MYC* |
| ERBB2 Regulates Cell Motility | 0,003484 | *ERBB2,RHOA* |
| Formation of the beta-catenin:TCF transactivating complex | 0,003636 | *MYC,CTNNB1* |
| RHO GTPase Effectors | 0,003828 | *CDC42,CTNNB1,PRKCA,RHOA,PAFAH1B1,MAPK3* |
| Signaling by WNT | 0,004059 | *MYC,CTNNB1,PRKCA,RHOA* |
| TFAP2 (AP-2) family regulates transcription of growth factors and their receptors | 0,004232 | *ERBB2* |
| PI3K events in ERBB2 signaling | 0,004631 | *PIK3CA,ERBB2* |
| Gastrin-CREB signalling pathway via PKC and MAPK | 0,005479 | *PRKCA,MAPK3* |
| Sema4D induced cell migration and growth-cone collapse | 0,006393 | *ERBB2,RHOA* |
| Signaling by ERBB2 ECD mutants | 0,006874 | *PIK3CA,ERBB2* |
| Signaling by TGF-beta Receptor Complex | 0,007006 | *MYC,RHOA* |
| Degradation of beta-catenin by the destruction complex | 0,008433 | *MYC,CTNNB1* |
| Myogenesis | 0,009515 | *CDC42,CTNNB1* |
| Sema4D in semaphorin signaling | 0,009515 | *ERBB2,RHOA* |
| MAPK family signaling cascades | 0,010115 | *CDC42,PIK3CA,MYC,ERBB2,MAPK3* |
| Platelet activation, signaling and aggregation | 0,011328 | *CDC42,PIK3CA,PRKCA,RHOA,MAPK3* |
| RHO GTPases activate IQGAPs | 0,011903 | *CDC42,CTNNB1* |
| SHC1 events in ERBB2 signaling | 0,011903 | *ERBB2,PRKCA* |
| MAPK6/MAPK4 signaling | 0,012703 | *CDC42,MYC* |
| RHO GTPases Activate NADPH Oxidases | 0,013186 | *PRKCA,MAPK3* |
| G-protein beta:gamma signalling | 0,013849 | *CDC42,RHOA* |
| CD28 co-stimulation | 0,013849 | *CDC42,PIK3CA* |
| SMAD2/SMAD3:SMAD4 heterotrimer regulates transcription | 0,013849 | *MYC* |
| RHO GTPases Activate WASPs and WAVEs | 0,015217 | *CDC42,MAPK3* |
| Signaling by TGFB family members | 0,015402 | *MYC,RHOA* |
| RET signaling | 0,016641 | *PIK3CA,PRKCA* |
| Transcriptional regulation by RUNX3 | 0,016864 | *MYC,CTNNB1* |
| Signaling by Rho GTPases | 0,018146 | *CDC42,CTNNB1,PRKCA,RHOA,PAFAH1B1,MAPK3* |
| Signaling by ERBB2 KD Mutants | 0,018879 | *PIK3CA,ERBB2* |
| PI5P, PP2A and IER3 Regulate PI3K/AKT Signaling | 0,020014 | *PIK3CA,ERBB2,MAPK3* |
| Signaling by ERBB2 in Cancer | 0,020437 | *PIK3CA,ERBB2* |
| Axonal growth stimulation | 0,022506 | *RHOA* |
| Resistance of ERBB2 KD mutants to afatinib | 0,022506 | *ERBB2* |
| Resistance of ERBB2 KD mutants to neratinib | 0,022506 | *ERBB2* |
| Resistance of ERBB2 KD mutants to tesevatinib | 0,022506 | *ERBB2* |
| Resistance of ERBB2 KD mutants to AEE788 | 0,022506 | *ERBB2* |
| Resistance of ERBB2 KD mutants to osimertinib | 0,022506 | *ERBB2* |
| Resistance of ERBB2 KD mutants to lapatinib | 0,022506 | *ERBB2* |
| Resistance of ERBB2 KD mutants to trastuzumab | 0,022506 | *ERBB2* |
| Drug resistance in ERBB2 TMD/JMD mutants | 0,022506 | *ERBB2* |
| Resistance of ERBB2 KD mutants to sapitinib | 0,022506 | *ERBB2* |
| EPHB-mediated forward signaling | 0,022872 | *CDC42,RHOA* |
| Signaling by FGFR4 | 0,022872 | *PIK3CA,MAPK3* |
| Negative regulation of the PI3K/AKT network | 0,023468 | *PIK3CA,ERBB2,MAPK3* |
| Signaling by Receptor Tyrosine Kinases | 0,024138 | *CDC42,PIK3CA,ERBB2,CTNNB1,PRKCA,RHOA,MAPK3* |
| Signaling by FGFR3 | 0,024558 | *PIK3CA,MAPK3* |
| Transcriptional activity of SMAD2/SMAD3:SMAD4 heterotrimer | 0,026294 | *MYC* |
| Diseases of signal transduction by growth factor receptors and second messengers | 0,026495 | *PIK3CA,MYC,ERBB2,CTNNB1,MAPK3* |
| PLCG1 events in ERBB2 signaling | 0,026947 | *ERBB2* |
| GRB7 events in ERBB2 signaling | 0,026947 | *ERBB2* |
| Signaling by MAP2K mutants | 0,026947 | *MAPK3* |
| TFAP2 (AP-2) family regulates transcription of cell cycle factors | 0,026947 | *MYC* |
| Signaling by NTRK1 (TRKA) | 0,027716 | *PIK3CA,RHOA,MAPK3* |
| NOTCH1 Intracellular Domain Regulates Transcription | 0,028078 | *MYC* |
| ESR-mediated signaling | 0,029516 | *PIK3CA,MYC,MAPK3* |
| RHO GTPases Activate Formins | 0,030762 | *CDC42,RHOA,PAFAH1B1* |
| LRR FLII-interacting protein 1 (LRRFIP1) activates type I IFN production | 0,031369 | *CTNNB1* |
| Signaling by EGFR | 0,031788 | *CDC42,PIK3CA* |
| Signaling by FGFR1 | 0,032744 | *PIK3CA,MAPK3* |
| Negative feedback regulation of MAPK pathway | 0,035770 | *MAPK3* |
| SLIT2:ROBO1 increases RHOA activity | 0,035770 | *RHOA* |
| Signaling by SCF-KIT | 0,036680 | *PIK3CA,PRKCA* |
| Constitutive Signaling by NOTCH1 PEST Domain Mutants | 0,038714 | *MYC* |
| Signaling by NOTCH1 PEST Domain Mutants in Cancer | 0,038714 | *MYC* |
| Signaling by NOTCH1 HD+PEST Domain Mutants in Cancer | 0,038714 | *MYC* |
| Constitutive Signaling by NOTCH1 HD+PEST Domain Mutants | 0,038714 | *MYC* |
| Signaling by NOTCH1 in Cancer | 0,038714 | *MYC* |
| Signaling by NTRKs | 0,040307 | *PIK3CA,RHOA,MAPK3* |
| Transcriptional regulation of granulopoiesis | 0,041842 | *MYC* |
| Signaling by Non-Receptor Tyrosine Kinases | 0,041842 | *ERBB2,RHOA* |
| Signaling by PTK6 | 0,041842 | *ERBB2,RHOA* |
| Semaphorin interactions | 0,042906 | *ERBB2,RHOA* |
| Insulin receptor signalling cascade | 0,042906 | *PIK3CA,MAPK3* |
| Gene and protein expression by JAK-STAT signaling after Interleukin-12 stimulation | 0,043979 | *CDC42* |
| Disinhibition of SNARE formation | 0,044514 | *PRKCA* |
| RHO GTPases Activate Rhotekin and Rhophilins | 0,044514 | *RHOA* |
| Activated NTRK3 signals through PI3K | 0,044514 | *PIK3CA* |
| MET activates PI3K/AKT signaling | 0,044514 | *PIK3CA* |
| Activated NTRK2 signals through PI3K | 0,048856 | *PIK3CA* |
| Axonal growth inhibition (RHOA activation) | 0,048856 | *RHOA* |
| ROBO receptors bind AKAP5 | 0,048856 | *PRKCA* |
| RSK activation | 0,048856 | *MAPK3* |
| Apoptotic cleavage of cell adhesion proteins | 0,048856 | *CTNNB1* |
